# Roles of miRNAs and long noncoding RNAs in the progression of diabetic retinopathy

**DOI:** 10.1042/BSR20171157

**Published:** 2017-11-29

**Authors:** Qiaoyun Gong, Guanfang Su

**Affiliations:** Eye Center, The Second Hospital of Jilin University, #218 Ziqiang Street, Changchun, Jilin 130021, China

**Keywords:** diabetic retinopathy, dysregulation, long noncoding RNA, molecular mechanisms, microRNA

## Abstract

Diabetic retinopathy (DR) is the leading cause of blindness in working-age adults across the world. The pathogenesis of DR is multifactorial and the molecular mechanisms are still not fully understood. Accumulating evidence has demonstrated that noncoding RNAs (ncRNAs) may be aberrantly expressed and may play vital roles in the development of DR. Amongst ncRNAs, miRNAs and long ncRNAs (lncRNAs) are known for their regulatory functions. Here, we summarize the functions and mechanisms of known aberrantly expressed miRNAs and lncRNAs in DR. Additionally, a novel lncRNA–mRNA–miRNA network is included in this review. We highlight original studies that provide detailed data about the mechanisms of miRNAs and lncRNAs, their applications as diagnostic or prognostic biomarkers, and their potential therapeutic targets. In conclusion, this review will help us gain a better understanding of the molecular mechanisms by which miRNAs and lncRNAs perform their functions in DR, and provide general strategies and directions for future research.

## Introduction

Diabetes mellitus (DM), defined by a fasting plasma glucose value of ≥7.0 mmol/l (126 mg/dl) or by receiving medication for raised blood glucose, is increasingly common and prevalent across the world. Diabetes poses a well-recognized increased risk for cardiovascular disease, kidney failure, blindness, and lower-limb amputation [1]. Diabetic retinopathy (DR) is one of the most serious microvascular complications and is the leading cause of visual disability and blindness in working-age adults (20–65 years) [2]. DR stems from abnormal retinal blood vessels that are either non-proliferative (non-proliferative DR (NPDR)) or proliferative (proliferative DR (PDR)). Visual impairment results from edema affecting the central retina or macula (diabetic macular edema (DME)). Thus, DR is the most common vision-threatening complication of DM and is the prime cause of visual loss in diabetic patients [3,4].

The microvascular circulation of the retina is predominantly affected by DM, resulting in a range of structural changes. These changes ultimately cause increased permeability and altered proliferation of endothelial cells (ECs), edema, and abnormal neovascularization (NV) of the retina, leading to vision loss [5]. The pathogenesis of DR is extremely complicated, implicating numerous different mechanisms. A previous study has shown that consistently elevated blood glucose in the retina can cause oxidative stress [6], characterized by excess reactive oxygen species (ROS) activating the four canonical pathways that damage retinal cells and tissues [7]. Inflammation is another important factor contributing to the progression of DR in the early stage [8]. During this stage of diabetes, the enhanced secretion of proinflammatory cytokines renders the retina a sustained inflammatory environment, ultimately resulting in retinal microvascular abnormalities [9]. Neuronal dysfunction is also detected in the progress of DR [10]. DM affects neurones via altered metabolism, which leads to apoptosis of the retinal ganglion cells (RGC) and activation of glial cells. Metabolic alterations are likely to be mediated by oxidative stress, inflammation, and elevated levels of glutamate [11]. In summary, oxidative stress and enhanced production of inflammatory mediators, accompanied by neurodegeneration, contribute to the pathogenesis and complexity of DR. In addition to the metabolic changes, hyperglycemic insult also affects the enzymatic machinery responsible for epigenetic modifications; these modifications alter gene expression without affecting the DNA sequence [12]. Epigenetics could be one of the major contributing participants in the development and progression of DR, but the role of epigenetics in DR is still in its incipient stages. The epigenetic modifications, including DNA methylation, histone modifications, and miRNAs regulation, alter and aggravate the progression of DR inspite of the controlled glucose [13]. Overall, epigenetics is an emerging area, and the diabetic environment favors epigenetic modifications; the genes implicated in the pathogenesis of DR and enzymes responsible for epigenetic modifications and miRNAs are altered in the retina in diabetes, suggesting the role of these post-translational modifications in the progression of DR. These mechanisms may interact closely, creating a network that leads to pathologic changes in the retina, including disruption of vascular integrity, increased vascular permeability, and retinal NV.

Amongst the pathways participating in the pathogenesis and progression of DR, a large number of related protein-encoding genes have been identified and shown to play crucial roles in vascular changes. These factors include vascular endothelial growth factor (VEGF) [14], pigment epithelium derived factor (PEDF) [15], angiopoietin [16], and bone morphogenetic protein (BMP) [17]. In addition to protein-coding genes, and unlike the known RNA molecules that perform infrastructural and housekeeping roles (such as tRNAs, rRNAs, and snRNAs), recent data suggest that enormous numbers of so-called noncoding RNAs (ncRNAs), having little to no protein-coding potential, are expressed and function in the pathogenesis of DR [18–20]. ncRNAs are divided into distinctive classes mainly according to origin, structure, and biological functions. In recent decades, miRNAs, a group of small ncRNAs, have been broadly studied in the different pathways involved in the pathogenesis of DR. Moreover, a novel class of transcripts called long ncRNAs (lncRNAs), which are pervasively transcribed in the genome, is gaining attention because of their potential regulatory roles in the progression and prevention of DR.

In this review, we will focus on discussing miRNAs, lncRNAs, and their potential biological roles and underlying molecular mechanisms in the pathogenesis and development of DR. Finally, we will discuss the prospects of using miRNAs and lncRNAs as potential biomarkers and therapeutic targets for the treatment of DR. This review will help us gain a better understanding of the molecular mechanisms, by which miRNAs and lncRNAs perform their functions and interactions in DR, and will provide direction for future research.

## Roles of miRNAs in DR

### Biogenesis and functions of miRNAs

miRNAs are a group of short (approximately 21–23 nts long) and highly conserved sequences of endogenous RNAs that do not code for any protein. miRNAs modulate gene expression through transcriptional and post-transcriptional regulation, inducing mRNA degradation or inhibiting protein translation by binding to the seed region on the 3′-UTR of target genes. They are expressed in all human cell types and are involved in main biological processes such as cell growth, differentiation, and apoptosis [4,21,22].

The synthesis of miRNAs is a complex biological process involving several enzymes located in both the nuclear and cytoplasmic compartments of cells. The coding region for miRNAs may be embedded within the intronic genomic regions of both coding and noncoding genes or within exons. Similar to mRNAs, transcription occurs in the nucleus, mediated by RNA polymerase II, leading to the generation of primary miRNAs (pri-miRNAs). pri-miRNAs are long (>1000 nts), capped, polyadenylated primary transcripts that contain specific loop-stem structures called hairpins. In the canonical processing pathway, pri-miRNAs are recognized and cleaved by the protein complex Drosha/DGCR8 (DiGeorge syndrome critical region gene 8), resulting in shorter nucleotide sequences (70–100 nts) known as pre-miRNAs [23,24]. Once synthesized, pre-miRNAs are recognized by the nuclear–cytoplasmic shuttle protein Exportin-5 and are transferred to the cytoplasm. Then, pre-miRNAs are processed by the RNase III protein Dicer, leaving shorter (21–23 nts) double-stranded RNA sequences. These sequences contain the mature miRNA and its passenger strand. The guide strand is then joined to one of the Argonaute (Ago) proteins to establish the core of the silencing complex called the RNA-induced silencing complex (RISC); the passenger strand is discarded and degraded [25]. The mature RISC can bind the UTR, generally the 3′-UTR. The so-called ‘seed region’, spanning between nucleotides 2 and 8 of the 5′-end of the mature miRNA, binds a complementary sequence in the 3′-UTR of target mRNAs. The complementary sequence, positioned between the seed region and mRNA-binding sites on the 3′-UTR, is necessary for target recognition [21]. However, several studies have shown that miRNA targetting is not always limited to the 3′-UTR, with respect to the 5′-UTR of mRNAs. There are naturally occurring examples of miRNA down-regulating the expression of the corresponding mRNA in a seed-dependent manner [26]. Finally, miRNA–mRNA binding leads to translational inhibition or degradation of the target mRNA. It is clear that the ultimate biological effect on cells occurs via the repression of specific proteins involved in a particular biological pathway.

A single miRNA may modulate the expression of several mRNAs; conversely, more than 60% of mRNAs have predicted binding sites for multiple miRNAs, thus allowing concomitant interaction with multiple miRNAs [27]. It is, therefore, expected that the function and biogenesis of miRNAs can be regulated, because their alteration is associated with a wide range of human diseases, including chronic conditions.

### Dysregulated miRNAs in DR

As described above, miRNAs play vital roles in the proliferation, migration, and apoptosis of human cells, including retinal cells. It is not surprising that miRNAs have been reported to play an important role in regulating DR-related NV. An uncontrolled high level of blood glucose is the leading cause of type 2 DM and related complications. DR is a chronic and serious eye complication associated with DM, presenting microvascular and macrovascular changes. In the early stage of DR, human retinal ECs (HRECs) and retinal pigment epithelial (RPE) cells, which are components of the blood–retinal barrier (BRB), are affected and impaired by the adverse effects of high glucose (HG); this induces dysfunction of BRB and contributes to progression of DR [28–31]. To date, numerous studies have investigated the roles of miRNAs in DR, using HRECs and RPE cells as *in vitro* and *in vivo* models of streptozotocin (STZ)-induced diabetic retinas*.*

Kovacs et al. [32] identified a series of miRNAs whose expression is altered in the retinas and retinal ECs (RECs) of STZ-induced diabetic rats 3 months after the onset of diabetes; this provided the first insight into the roles of miRNAs in the pathogenesis of DR. miRNA array profiling has revealed that in retinas of diabetic rats, the expression of 80 miRNAs was significantly increased, and expression of 6 miRNAs was decreased, compared with that of controls. In the RECs of diabetic rats, 16 miRNAs were up-regulated (*P*<0.01) and 104 miRNAs were down-regulated compared with those in controls (*P*<0.01). Furthermore, the same study identified NF-κB-, VEGF-, and p53-responsive miRNAs. The up-regulation of *miR-146, miR-155, miR-132*, and *miR-21* in the RECs of diabetic rats is known to be NF-κB-responsive, suggesting an miRNA signature in NF-κB activation in diabetic RECs. Additionally, VEGF-responsive miRNAs (*miR-17-5p, miR-18a, miR-20a, miR-21, miR-31*, and *miR-155*) are up-regulated in both retinas and RECs of diabetic rats when the level of VEGF is increased. This implies that these miRNAs may be involved in the pathogenesis of DR partly through mediating functions of VEGF. The *miR-34* family, including *miR-34a/b/c*, is a direct transcriptional target of p53 and contributes to p53-mediated cell-cycle arrest, apoptosis, and senescence [33]. In the diabetic retina, the levels of p53-responsive *miR-34* family become elevated in response to the activation of p53. This suggests that the *miR-34* family plays a role in the p53-induced apoptosis in neuroretinal and ECs, thus, contributing to the progression of DR. Wu et al. [34] has reported a significantly altered expression of miRNAs in the retinas of STZ-induced diabetic rats 10 weeks after injection with STZ. Microarray profiles and real-time PCR analyses indicate that a total of 11 miRNAs (*miR-182, miR-96, miR-183, miR-211, miR-204, miR-124, miR-135b, miR-592, miR-190b, miR-363*, and miR-29c*) show increased expression, whereas 6 miRNAs (*miR-10b, miR-10a, miR-219-2-3p, miR-144, miR-338*, and *miR-199a-3p*) are found at decreased levels, in the retinas of DM rats compared with those in normal controls. The variation in the expression levels of some retinal miRNAs parallels the course of DR, suggesting a close association between miRNA dysregulation and the development of DR. Xiong et al. identified 17 dysregulated miRNAs, in the retinas of STZ-induced DM rats, 10 weeks after the onset of DM [35].GO analysis revealed that the most prevalent miRNAs are related to olfactory bulb development, axonogenesis, and mitogen-activated protein and calcium signaling pathways. An additional six miRNAs (*miR-203, -350, -212, -216a, -410*, and *-34c*) were further validated by RT-qPCR. These six miRNAs are at the core of a network and most likely to have the most significant regulatory functions [35]. The expression levels of *miR-216a-5p, -34c-5p, -410-3p*, and *-203a-3p* are significantly up-regulated in the DM group compared with that in the control group, while *miR-212-3p* is down-regulated, and *miR-350* shows no significant change. Additionally, in our recent study, we identified several miRNAs, both in HRECs and ARPE-19 cells, whose levels increase with time under hyperglycemia; the levels of these miRNAs also increase in the retinas of STZ-induced DM rats for 4, 6, and 8 weeks after STZ injection [36]. Amongst the identified miRNAs, *miR-124-3p, miR-125b-5p, miR-135b-5p*, and *miR-199a-5p* showed a gradually decreased expression in HRECs and RPE cells; this paralleled an increase in hyperglycemia-treatment time. *miR-145-5p* and *miR-146a-5p* exhibited the opposite changes in expression (down-regulation in HRECs and up-regulation in RPE cells) in response to HG exposure over increasing durations. In the retinas of DM rats, *miR-124-3p* and *miR-125b-5p* remain down-regulated during the different stages of DR, while *miR-135b-5p, miR-145-5p, miR-146a-5p*, and *miR-199a-5p* show increased expression that parallels the progression of DR. These findings imply that the same miRNAs may vary, and play opposite roles, during DR development. The underlying reason for these differences can be explained by the fact that the pathological changes, associated with DR progression, and the intrinsic variability of DR etiogenesis, exert distinct effects on miRNA biogenesis, resulting in either up-regulation or down-regulation at different stages of DR. Much of this discrepancy may also be due to different sample sizes and different laboratory techniques.

### Specific miRNAs and their targets in different pathways involved in DR

#### miR-200b

*miR-200b* has been the most studied miRNA since it was first discovered. In ECs, treated with HG and in the retinas of STZ-induced diabetic rats 1 month after the onset of diabetes, the expression of *miR-200b* is decreased, whereas its validated direct target, VEGF, is increased at both the mRNA and protein levels [37]. Overexpression of *miR-200b* ameliorates VEGF expression in the retinas of diabetic rats and prevents glucose-induced increased permeability and angiogenesis in human umbilical vein ECs (HUVECs). Conversely, an *miR-200b* antagonist can increase VEGF production, further demonstrating the role of this miRNA in the pathogenesis of DR. However, another study, performed in a genetic model of type 1 diabetes (Akita mice), found that the expression of *miR-200b* increases significantly in 8-month-old diabetic rats compared with that in controls [38]. Increased expression of *miR-200b* inhibits the expression of oxidation resistance 1 (*Oxr1*), a protective gene involved in resistance to apoptosis and oxidative stress. Moreover, the level of *miR-200b* is decreased in HRECs in an HG environment, and is accompanied by increased levels of VEGF and transforming growth factor β (TGF-β) 1 (TGF-β1). *miR-200b* regulates the proliferation of HRECs by altering VEGF and TGF-β1 expression, thus delaying DR [39]. The levels of *miR-200b* and *VEGFA* mRNA, in the sera of DR patients, indicate that DR patients show decreased expression of *miR-200b* and increased *VEGFA* mRNA in comparison with those in healthy people [40]. Another study found that the expression of *miR-200b* is increased in the vitreous samples from eyes with PDR compared with those of controls (*P*<0.001). Vitreous VEGF expression is also significantly higher in the PDR group than in the control group (*P*<0.001); however, no significant correlation is found between *miR-200b* and VEGF [41]. According to these studies, *miR-200b* is down-regulated in ECs under HG, in the retinas of STZ-induced diabetic rats and in the sera of DR patients, whereas it is up-regulated in the retinas of Akita mice and vitreous of PDR patients. These discrepancies in the expression of *miR-200b* may have multiple causes. The first cause may be due to the use of different diabetic models, including cultured retinal cells, STZ-induced DM rats, the Akita mouse model, and serum or vitreous samples from DR patients. Another reason may be due to differences in the duration of diabetes, which may also contribute to the variability in the results. Additionally, the pathological changes, associated with DR progression, may exert distinct effects on miRNA biogenesis, resulting in either up-regulation or down-regulation at different stages of DR.

#### miR-146

*miR-146* was first implicated in DR when its roles were investigated in the modulation of the NF-κB inflammatory response. NF-κB is a ubiquitous inducible transcription factor and key modulator of cellular inflammatory pathways, playing a role in the early phases of DR development [42]. The increased level of *miR-146a*, in the RECs of diabetic rats, is transactivated by NF-κB. The increased level of *miR-146a* also exerts negative feedback on IL-1R/toll-like receptor (TLR) mediated NF-κB activation by targetting IL-1 receptor associated kinase 1 (IRAK1) and tumor necrosis factor (TNF) receptor associated factor 6 (TRAF6) [32]. IRAK1 and TRAF6 are downstream members of the myeloid differentiation primary-response protein-88 (MyD88)-dependent IL-1R/TLR-mediated NF-κB activation pathway in monocytes [43]. Cowan et al. [44] has uncovered a novel negative feedback regulatory mechanism, involving thrombin-induced and G-protein-coupled receptor (GPCR) mediated activation of NF-κB by *miR-146*, which targets caspase-recruitment domain (CARD) containing protein 10 (CARD10) in HRECs. Recently, Ye et al. demonstrated that the expression of *miR-146a* was down-regulated in HRECs cultured in HG 45[]. Overexpression of *miR-146a* reduced the levels of TLR4/NF-κB and TNF-α, and inhibited the MyD88-dependent and -independent pathways in RECs, under hyperglycemic conditions [45]. These results indicate that *miR-146a* can play a negative regulatory role in multiple pathways of NF-κB activation, which are related to inflammatory processes in DR. Furthermore, *miR-146a* reduces the expression of NF-κB-responsive adhesion molecule intercellular adhesion molecule-1 (ICAM-1) by binding to the 3′-UTR of IRAK1, an upstream adapter protein activated by IL-1β in both HRECs and diabetic rat retinas [46]. *miR-146a* and IRAK1 exhibit daily oscillations in antiphase; however, these patterns are lost in the diabetic retina. The loss of the rhythmic pattern is associated with an increase in ICAM-1, IL-1β, and VEGF expression, and contributes to the progression of DR. A following study, performed *in vivo* to confirm the regulatory role of *miR-146a* in the NF-κB-responsive proinflammatory pathway, has shown that intravitreal injection of lenti-*miR-146a* increases the expression of *miR-146a* in the retina, while its key downstream target genes, including CARD10, IRAK1, and TRAF6, are down-regulated [47]. Intravitreal delivery of *miR-146* inhibits the diabetes-induced up-regulation of the NF-κB downstream gene *ICAM1*, microvascular leakage, and retinal functional defects. These results indicate that *miR-146* may be an alternative therapeutic target for the treatment of DR via inhibition of NF-κB. Moreover, a decrease in the level of *miR-146a*, and the overexpression of fibronectin (FN), are observed in HG-treated ECs and retinas of STZ-induced diabetic rats at 1 month of DM [48]. FN is an extracellular matrix (ECM) protein, and its increased production is a characteristic feature of all chronic complications in diabetes. *miR-146a* can regulate FN directly by binding to the 3′-UTR of FN; therefore, the up-regulation of *miR-146a* may be a key mechanism for ameliorating the increased production of ECM proteins in diabetes. Recently, another pathway, involving miR-146a, indicated that *miR-146a* is down-regulated in HRECs under HG. However, elevated expression of *miR-146a* resulted in inhibition of signal transducer and activator of transcription 3 (STAT3) and VEGF signaling via down-regulation of IL-6 in HRECs under HG conditions. This suggests that *miR-146a* can protect HRECs from HG-induced apoptosis by suppressing the STAT3/VEGF pathway via IL-6 signaling [49].

The association of *miR-146a* with inflammation in DR may also involve *miR-146b*. Anti-inflammatory processes may be induced by extracellular adenosine that activates adenosine receptors. Extracellular adenosine is regulated by the interplay of the equilibrative nucleoside transporter with adenosine deaminase (ADA) and adenosine kinase. In the vitreous of diabetic patients, decreased *miR-146b-3p* is associated with increased ADA2 activity. Elevated expression of *miR-146b-3p* suppresses the expression and activity of ADA2, and TNF-α release, in amadori-glycated albumin (AGA) treated human macrophages. These results suggest that by suppressing ADA2, *miR-146b-3p* plays a regulatory role in diabetes-related retinal inflammation [50]. A following study investigated the role of *miR-146b-3p* in HRECs. They found that *miR-146b* reverses the changes in AGA-treated macrophages, significantly increasing HREC permeability, rescuing the disrupted pattern of zonula occludens (ZO)-1, and increasing leukocyte adhesion to HRECs by up-regulating ICAM-1. Therefore, inhibition of ADA2 by *miR-146b-3p* may be a useful tool for preserving the BRB function in DR [51].

#### miR-126

Down-regulation of *miR-126* is observed in the retinas of oxygen-induced retinopathy (OIR) mice. Retinal NV is a key pathological alteration in PDR, leading to dramatic vision loss; it is mediated by numerous angiogenic factors such as VEGF, insulin/insulin-like growth factor (IGF), and hypoxia-inducible factor-1 α (HIF-1α). The restoration of *miR-126* levels ameliorates the high levels of VEGF, IGF, and HIF-1α, resulting in reduction in retinal NV under ischemic conditions. The production of angiogenic factors, regulated by *miR-126*, may depend on p38 and extracellular signal regulated kinase (ERK), both enzymes of the mitogen-activated protein kinase (MAPK) pathway. In the ischemic retina, reduced levels of *miR-126* may stimulate the activation of p38 and ERK pathways, and increase the expression of angiogenic factors downstream [52]. Ye et al. has confirmed the down-regulation of *miR-126* in hypoxia-induced RF/6A cells *in vitro* and in diabetic retinas of STZ-induced diabetic rats 3 months after the onset of diabetes. Restoration of *miR-126* expression halts the hypoxia-induced NV by suspending cell-cycle progression and inhibiting the expression of VEGF and matrix metalloproteinase 9 (MMP-9) [53]. Furthermore, *miR-126* is down-regulated in human retinal pericytes under conditions of intermittent HG. Extracellular vesicles (EVs), derived from mesenchymal stem cells (MSCs) maintained under diabetes-like conditions, may play a role in vessel destabilization, thus contributing to angiogenesis through paracrine signaling. MSC-derived EV, obtained under hyperglycemic/hypoxic conditions, down-regulate the expression of *miR-126* in pericytes, leading to increased expression of angiogenic molecules such as VEGF and HIF-1α. These results suggest that *miR-126* potentially plays a role in EV-induced vessel destabilization in DR [54]. Clinically, a significant decrease in the serum expression of *miR-126* is detected in diabetic patients with complications (especially those with diabetic macrovascular complications and DR) compared with the levels of those without evident complications. Serum expression of *miR-126* may be a good marker for diagnosing type 2 DM and for monitoring the outcomes of this disease [55]. Similarly, in a large cohort of type 1 diabetic patients, the levels of *miR-126* were significantly lower than those in controls, and were associated with vascular complications of diabetes, particularly with proliferative retinopathy [56].

#### Other miRNAs

Up-regulation of *miR-29b* was first detected in the retinas of diabetic rats within 35 days after STZ injection. Proapoptotic RNA-dependent protein kinase (PKR) associated protein X (RAX) is indirectly regulated by *miR-29b* [57]. *miR-29b* and RAX are localized in the RGCs and the cells of the inner nuclear layer (INL) in the retinas of normal and STZ-induced diabetic rats. The up-regulation of *miR-29b*, in the early stage of STZ-induced DM rats, may have a protective effect against apoptosis in RGCs and cells of the INL via the proapoptotic PKR pathway. Moreover, in an *in vitro* study, Lin et al. observed that *miR-29* is up-regulated by HG in RPE cells [58]. Down-regulation of *miR-29* protects against HG-induced apoptosis via direct targetting of phosphatase and tensin homolog (PTEN), and reduced production of caspase-7 in RPE cells [58]. *miR-29a* is decreased in diabetic retinas with overexpressed angiotensinogen (AGT). Up-regulation of *miR-29a* inhibits the high levels of AGT and significantly ameliorates the vascular density, tortuosity, and EC nuclei [59]. AGT is part of the renin–angiotensin system, which exists in various organs, participates in the occurrence and progression of a variety of diseases, and is linked with diabetes [60]. Therefore, *miR-29a* may prevent the development of DR in the rat model via down-regulation of AGT.

Mortuza et al. [61] demonstrated that up-regulation of *miR-195* in HRECs exposed to HG and in the retinas of diabetic rats is accompanied by a concomitant down-regulation of silent information regulator protein 1 (SIRT1). Glucose-induced increased oxidative stress causes rapid aging in ECs and retinas in diabetes; these processes are mediated via alteration of SIRTs. Neutralization of *miR-195* rescues SIRT1 expression directly and decreases tissue damage in DR, highlighting a potential role of *miR-195*-SIRT1 signaling in modulation of REC function. Consistent with these findings, oxidative stress induced overexpression of *miR-195* causes direct down-regulation of mitofusin 2 (MFN2) in HRECs and diabetic retinas, leading to tube formation and increased permeability of retinal BRB, which are two common pathogenic functional changes associated with DR [62]. MFN2 is a multifunctional mitochondrial membrane protein associated with oxidative stress and diabetes-related complications [63]. Therefore, *miR-195* may be a potential therapeutic target because it regulates molecules associated with oxidative stress in the progression of DR. SIRT1 is also a direct target of *miR-23b-3p*, which is elevated by HG in HRECs. Reduced expression of *miR-23b-3p* inhibits the expression of acetylated NF-κB by rescuing the expression of SIRT1, and relieves the effect of metabolic memory induced by HG, in HRECs. These results were confirmed in the retina of a diabetic rat model of metabolic memory. Therefore, *miR-23b-3p* regulates HG-induced cellular metabolic memory in DR through a SIRT1-dependent signaling pathway [64].

Kovacs et al. [32] identified an increased level of the *miR-34* family in the retinas and RECs of diabetic rats *in vivo*. However, Hou et al. confirmed the down-regulation of *miR-34a* in subconfluent ARPE-19 cells [65]. The proliferative and migratory phenotype of RPE cells occur under pathological conditions such as DR. Up-regulation of *miR-34a* can inhibit the proliferation and migration of RPE cells by inhibiting its target, c-Met, and other cell cycle related molecules such as CDK2, CDK4, CDK6, E2F1, and p-Cdc2 [65]. Furthermore, up-regulation of *miR-34a* inhibits the proliferation, migration, and attachment of RPE cells, partly by down-regulating the expression of leucine-rich repeat containing GPCR 4 (LGR4) and inducing reduced expression levels of cell cycle mediators E2F1, p-CDC2, CDK2, CDK4, and CDK6 [66].

The expression of *miR-15b* and *miR-16* is reduced in HRECs cultured under hyperglycemic conditions for 3 days. Overexpression of *miR-15b* and *miR-16* inhibits the levels of TNF-α and suppressor of cytokine signaling 3 (SOCS3), while increasing that of IGF-binding protein-3 (IGFBP-3) and the phosphorylation of insulin receptor (IR) Tyr^1150/1151^. This shows that *miR-15b* and *miR-16* play a role in the inhibition of insulin resistance, resulting in HREC protection from hyperglycemia-induced apoptosis [67]. In addition to investigating the regulatory effects of *miR-15a* and *miR-16* on insulin resistance, Ye et al. has confirmed that *miR-15a* and *miR-16* play inhibitory roles in proinflammatory signaling, reducing retinal leukostasis in DR [68]. Hyperglycemia induces the down-regulation of *miR-15a* and *miR-16*, while overexpression of *miR-15a* and *miR-16* significantly decreases proinflammatory signaling of IL-1β, TNF-α, and NF-κB in HRECs under conditions of HG. An *in vivo* study, using knockout mice in which *miR-15a* and *miR-16* are eliminated in the vascular ECs, has shown that the loss of *miR-15a* and *miR-16* leads to increased retinal leukostasis and levels of CD45, as well as up-regulated levels of IL-1β, TNF-α, and NF-κB. Therefore, *miR-15a* and *miR-16* may play significant roles in reducing retinal leukostasis, potentially through inhibition of inflammatory cellular signaling [68]. Wang et al. has reported on the dual anti-inflammatory and anti-angiogenic actions of *miR-15a* in DR [69]. *miR-15a* is reduced in the retina and bone marrow cells in diabetes. Inhibition of *miR-15a* up-regulates the expression of acid sphingomyelinase (ASM), a proinflammatory molecule, and that of VEGFA, an angiogenic molecule, in RPE and ECs; this is accompanied by impairment of migration and retinal vascular repair function. In the animal model of overexpressed miR-15a, ASM and VEGFA levels are directly reduced to nondiabetic levels; diabetes-induced increased retinal permeability is also prevented in these mice [69]. These studies indicate that *miR-15* may be modulating multiple pathologies in DR.

The up-regulation of *miR-21* and *miR-155* is NF-κB responsive in RECs and retinas of diabetic rats [32]. Another study found that inhibition of *miR-21* significantly enhances the HG-induced endothelial cytotoxicity. Further, overexpression of *miR-21* inhibits the expression of death domain associated protein (DAXX), a proapoptotic mediator, whereas silencing DAXX mRNA reverses the inhibitory effects of *miR-21* on HG-induced endothelial apoptosis; this indicates that *miR-21* can protect ECs from apoptosis by inhibiting the expression of DAXX [70]. Recently, it was found that increased levels of *miR-21* in the vitreous are associated with retinal fibrosis, including PDR and proliferative vitreoretinopathy (PVR). The expression of *miR-21* in RPE cells, under HG conditions, is enhanced and induced by TGF-β, suggesting that its role positively correlates with disease progression. Furthermore, gain- and loss-of-function studies have revealed that *miR-21* promotes proliferation and migration of ARPE-19 cells. The present study indicates that *miR-21* is a potential disease-modifying miRNA in the vitreous humor and is involved in the development of retinal fibrosis [71]. *miR-155* is involved in immunomodulatory signaling [72]. The expression levels of *miR-155* in the peripheral blood of patients with PDR are significantly increased compared with those in the negative control (NC) group; the percentage of Treg cells, and the expression levels of TGF-β, are significantly decreased in PDR patients. The expression of *miR-155* is negatively correlated with the amount of Treg cells and the expression level of TGF-β, suggesting that *miR-155* may play a role in the pathogenesis of T2DM retinopathy [73].

The levels of a number of growth and vasoactive factors, especially VEGF, are increased in response to hyperglycemia. Dysfunction of ECs is the primary cellular pathology in complications of chronic diabetes. ECs undergo functional alterations that cause NV in response to VEGF. Hence, VEGF-mediated alterations are of significance in the early (vascular permeability), as well as late (NV), stages of DR. An increasing number of miRNAs are discovered and investigated in the regulation of VEGF in DR. Ling et al. [74] demonstrated a cross-talk between HIF-1α and VEGF via interactions with 12 common miRNAs in ARPE-19 and RPE cells isolated from mice. Silencing HIF-1α or VEGF increases the availabilities of shared miRNAs. Up-regulation of a common miRNA (*miR-106a*) significantly decreases the expression of HIF-1α and VEGF, and prevents HG-induced increased permeability, in an *in vivo* animal model of type 1 DM. Because VEGF and HIF-1α play important roles in the progression of DR and other retinal diseases, such cross-talk may have significant therapeutic implications [74]. Similarly, miRNA-dependent cross-talk between VEGF and angiopoietin-2 (Ang-2) was investigated. Ang-2 and VEGF are involved in the regulation of angiogenesis and are commonly regulated by *miR-351. miR-351*-dependent cross-talk between VEGF and Ang-2 occurs in hypoxic RF/6A cells and in rat retinas. Overexpression of *miR-351* significantly reduces the high levels of VEGF and Ang-2 *in vitro* and in an *in vivo* OIR animal model*.* Overall, *miR-351*-dependent cross-talk between Ang-2 and VEGF plays a role in the microvascular response induced by hypoxia [75]. Further, VEGF can be modulated by RAC-γ serine/threonine-protein kinase 3 (AKT3), which is targetted by *miR-20b* in DR. *miR-20b* is down-regulated, while AKT3 and VEGF are elevated in HRECs under HG conditions. Overexpression of *miR-20b* inhibits AKT3, and the silencing of AKT3 causes a decrease in the levels of VEGF, preventing an HG-induced increase in transendothelial permeability and tube formation. The regulating effect of *miR-20b*, on the mediation of VEGF by AKT3, has been confirmed *in vivo* in STZ-induced diabetic rats. This indicates that *miR-20b* directly targets AKT3 and modulates VEGF-mediated changes in DR [76]. Wu et al. found that the decrease in *miR-18b*, in HG-induced HRECs, promotes cell proliferation and production of VEGF [77]. Further, *miR-18b* performs its function by targetting IGF-1 directly. Stimulation of IGF-1 antagonizes the effect induced by up- regulation of miR-18b, *promoting cell proliferation and increased VEGF production. The opposite is* observed upon silencing of IGF-1, which is consistent with the effects of *miR-18b* overexpression. *miR-18b* exerts its function on VEGF synthesis and cell proliferation by suppressing the IGF-1 receptor (IGF1R) pathway, consequently inhibiting the downstream molecules. Thus, *miR-18b* can attenuate the expression of VEGF, and proliferation of HG-induced HRECs, by suppressing the IGF-1/IGF1R signaling pathway; this has potential clinical significance [77].

Genes of the *let-7* family are the founding members of miRNAs, with established functions in tissue differentiation and tumor suppression [78,79]. The role of the *let-7* gene family in angiogenesis, related to DR and age-related macular degeneration (AMD), was first investigated by Zhou et al. [80]. The *let-7* family genes are expressed in HRECs, choroidal ECs, and ARPE-19. Overexpression of *let-7 in vivo* leads to features of NPDR, including tortuous retinal vessels and defective pericyte coverage; however, this phenotype does not progress to PDR. Overexpression of *let-7* represses the proliferation, migration, and networking of ECs, while inhibition of *let-7* has the opposite effect. Moreover, *let-7* transgenic mice exhibit significantly reduced NV in the choroid after laser injury, while inhibition of *let-7*, using the *let-7* anti-miR, strongly enhances choroidal NV (CNV). The study indicates that *let-7* contributes to NPDR, but represses angiogenesis and CNV, *in vivo*.

### Aberrant miRNAs as biomarkers in serum and vitreous humor of patients with DM

Circulating miRNAs have emerged as novel biomarkers of diabetes. Cells release miRNAs into the circulation or their surrounding environment, where miRNAs have a long lifespan of more than 2 weeks. Hirota et al. [81] first investigated the expression of 168 miRNAs in the vitreous humor and serum samples of patients with PDR and macular hole. This study found that six miRNAs (*miR-15a, miR-320a, miR-320b, miR-93, miR-29a*, and *miR-423-5p*), related to angiogenesis and fibrosis, are significantly overexpressed in PDR [81]. Zampetaki et al. [81] evaluated 300 serum samples from two DR-related randomized, double-blind, parallel-design, placebo-controlled clinical trials (PROTECT-1 and PREVENT-1). They assessed 155 baseline and DR progressors, and 145 control samples (selected from 3326 study participants), for a panel of 29 candidate miRNAs that were based on previous studies related to diabetes and myocardial infarction [82,83]. They identified *miR-27b* and *miR-320a* as being significantly and independently associated with high DR risk. They also elucidated the potential mechanism, using cultured human ECs, and confirmed anti-angiogenic thrombospondin-1 as common target of these two miRNAs. The study identified *miR-320a* and *miR-27b* as potential biomarkers for DR [84]. Further, Usui-Ouchi et al. [71] explored the expression of 377 miRNAs in the vitreous humor of patients with proliferative vitreoretinal disease (PVD). They found that 23 miRNAs are consistently up-regulated in the PVD group, whereas 12 miRNAs are consistently down-regulated. A volcano plot, filtering against these miRNA expression profiles, identified the expression levels of five miRNAs, including let-7e, *miR-204, miR-216b, miR-9*, and *miR-139-5p* as significantly down-regulated in the PVD group. The expression levels of nine miRNAs, such as *miR-16, miR-92a, miR-130b, miR-21, miR-320*, and *miR-106b*, are significantly up-regulated in the PVD group. Gomaa et al. [41] found that the expression of *miR-200b* is increased approximately five-fold in the vitreous samples from eyes with PDR compared with those of controls, while VEGF expression is also significantly higher in the PDR group. No significant correlation is found between *miR-200b* and VEGF [41]. Thus, further studies are needed to understand the roles, played by specific miRNAs, in the biological function of the eye.

## Roles of lncRNAs in DR

### The biology of lncRNAs

In recent years, advancements in genome-wide analyses of the mammalian transcriptome have revealed a novel class of transcripts, lncRNAs, which are pervasively transcribed in the genome [85]. lncRNAs stand for a class of RNA molecules that are fundamentally different from miRNAs in their functions and actions. Unlike the short sequences of miRNAs, the transcripts for lncRNAs are usually longer than 200 nt and can even reach more than 100 kb [86]. The transcripts for lncRNAs lack significant ORFs and are localized in both the nucleus and cytoplasm [87,88]. Similar to protein-coding genes, the transcription of lncRNAs can be regulated, showing specific temporal and cell-type-specific expression [89,90]. The majority of lncRNAs are transcribed from the nuclear, or less frequently, from the mitochondrial, genome. The primary transcript can also be modified post-transcriptionally, similar to conventional mRNA modifications, including 5′ capping, 3′ polyadenylation, and splicing [85,87]. As a result of their prolonged length and sequence, the transcripts of lncRNAs can form specific secondary structures with clear functional features [91,92]. They modulate cellular gene expression at multiple levels and may act as signals, decoys, guides, and scaffolds [93]. lncRNAs may guide transcription factors to, or sequester them from, a specific region of action, or they may interact with multiple components, thereby repressing or activating gene expression [94]. Through intricate mechanisms, they play significant roles in various biological processes, including cell differentiation, apoptosis, nuclear trafficking, heat shock response, and more. An increasing number of studies have identified lncRNAs as a new class of modulatory molecules that participate in various human diseases by regulating gene expression at the transcriptional, post-transcriptional, or epigenetic level [95]. lncRNAs can induce or reduce protein translation via alternative splicing, turnover, export, and translocation of mRNAs. They can reduce the effect of miRNAs on mRNA stability by acting as competing endogenous RNAs (ceRNAs) or RNA sponges when they contain an miRNA-binding sequence region. Thus, the role of lncRNAs, in the pathogenesis of DR, deserves to be explored and investigated. Here, we review the aberrant lncRNAs and summarize the specific identified lncRNAs in DR (Table 1).

**Table 1:** Dysregulated lncRNAs involved in DR

LncRNA	Dysregulation	Tissues and cell type	Pathogenic functions	Possible mechanism	References
MIAT	Up-regulated	Retinas of DM rats; RF/6A, RPE, RGC and Müller cells	Promotes EC proliferation, migration, and tube formation *in vitro*	NF-κB activation	[96–98]
			Aggravates retinal vessel impairments and visual dysfunction *in vivo*		
MALAT1	Up-regulated	Retinas of DM mice; RF/6A	Promotes EC proliferation, migration, and tube formation *in vitro*	Cross-talks with p38 MAPK signaling pathway	[99–101]
			Deteriorates retinal NV *in vivo*	Activates inflammatory pathway via TNF-α and IL-6	
MEG3	Down-regulated	Retinas of DM mice; RF/6A	Regulates ECl proliferation, migration, and tube formation *in vitro*	Activates PI3K-Akt signaling pathway	[102]
			Aggravates retinal vessel dysfunction *in vivo*		
SOX2OT	Down-regulated	Retinas of DM mice; RGCs	Decreases cell viability and increases cell apoptosis *in vitro*	Antioxidative via regulation of NRF2/HO-1 signaling activity	[103]
			Promotes neurodegeneration *in vivo*		
RNCR3	Up-regulated	Retinas of DM mice; RF/6A, Müller cells	Increases cell viability and proliferation, promotes EC migration and tube formation *in vitro*	Related to the release of several cytokines	[104,105]
			Aggravates retinal cell apoptosis, visual function, and microvascular leakage *in vivo*		
ANRIL	Up-regulated	Retinas of DM mice; HRECs	Increases retinal microvascular permeability *in vivo*	Increases VEGF mediated by PRC2 complex and p300	[106]
BDNF-AS	Up-regulated	ARPE-19	Increases cell apoptosis *in vitro*	Not only via BDNF-associated pathways	[107,108]

Abbreviations: ANRIL, antisense RNA to INK4 locus; BDNF-AS, brain-derived neurotrophic factor antisense; MALAT1, metastasis-associated lung adenocarcinoma transcript 1; MEG3, maternally expressed gene 3; MIAT, myocardial infarction associated transcript; NRF2/HO-1, nuclear factor erythroid 2 related factor 2/heme oxygenase-1; PRC2, polycomb repressor complex 2; RNCR2, retinal ncRNA 2; SOX2OT, SOX2 overlapping transcript.

### Aberrant expression of lncRNAs in DR

The profiling of lncRNA expression in the retinas of STZ-induced diabetic mice was first performed using microarray analysis by Yan et al. [20]. In this study, approximately 303 lncRNAs were differently expressed in the retinas with early DR; these included 214 down-regulated lncRNAs and 89 up-regulated lncRNAs. GO analysis indicated that these lncRNAs coexpressed mRNAs are related to the development of the eye, integral to membrane and structural molecular activity. Pathway analysis indicated that lncRNAs coexpressed mRNAs are involved in several pathways, including axon guidance, MAPK signaling pathway, complement and coagulation cascades, chemokine signaling pathway, and pyruvate metabolism. These signaling pathways are closely associated with pathological processes, such as neurodegeneration, NV, inflammation, and immunology, suggesting that the lncRNA-mediated network plays an extensive role in the pathogenesis of DR. Furthermore, metastasis-associated lung adenocarcinoma transcript 1 (MALAT1), a highly conserved lncRNA, is significantly overexpressed in RF/6A cells under hyperglycemic conditions, as well as in aqueous humor samples and fibrovascular membranes of diabetic patients. This implies that lncRNAs are involved in the pathogenesis of DR via modulation of multiple pathogenic pathways.

A three-stage genome-wide association study (GWAS) was conducted to identify susceptible long intergenic ncRNAs in DR amongst the Japanese population [109]. A total of 837 type 2 diabetes patients with DR (cases) and 1149 individuals without DR (controls) were enrolled in the study. After the three-stage GWAS, the top signal, found in this association analysis, was rs9362054 in an intron of RP1-90L14.1, showing borderline genome-wide significance. While RP1-90L14.1 is a long intergenic ncRNA (lincRNA), adjacent to the *KIAA1009/QN1/CEP162* gene, CEP162 plays a critical role in the formation of the ciliary transition zone before ciliogenesis. The present study raised the possibility that the dysregulation of ciliary-associated genes plays a role in susceptibility to DR.

### Potential functional lncRNAs in DR

#### MIAT

Myocardial infarction associated transcript (MIAT), also known as Gomafu or retinal ncRNA 2 (RNCR2), was first indicated as a susceptibility locus for myocardial infarction [96] and found to be highly expressed in retinal precursor cells [97]. Yan et al. [98] has shown that MIAT expression is strongly up-regulated in the retinas of diabetic rats and in FVMs from PDR patients. Consistently, HG conditions induced high levels of MIAT in RF/6A ECs, RPE cells, RGCs, and Müller cells *in vitro.* In turn, knockdown of MIAT improves visual functions, under diabetic conditions, by inhibiting apoptosis. MIAT down-regulation alleviates retinal vessel impairment, *in vivo*, by reducing vascular leakage and counteracting the DM-induced up-regulation of proinflammatory proteins such as ICAM-1, TNF-α, and VEGF. MIAT knockdown *in vitro*, in ECs, inhibits EC proliferation, migration, and tube formation under diabetic conditions. Because MIAT is involved in the different stages of DR, MIAT knockdown is therapeutically effective against neovascular diseases [110]. MIAT is overexpressed in Müller cells, isolated from STZ-induced DM mice, and in rat retinal Müller cells (rMC-1) with HG; this is accompanied by the up-regulation of p-p65, indicating increased activity of NF-κB. ChIP results have revealed that HG promotes the binding activity between NF-κB and MIAT, indicating that NF-κB selectively binds to the MIAT promoter [99].

#### MALAT1

MALAT1 is highly expressed in different kinds of tumors, including lung cancer, liver cancer, renal cell carcinoma, bladder cancer, and osteosarcoma; it also participates in the pathogenesis of DR [100]. MALAT1 is significantly up-regulated in the retinas of diabetic mice, in HG-treated RF/6A cells, and in the aqueous humor samples and fibrovascular membranes of diabetic patients [20]. Liu et al. found that MALAT1 regulates REC function and pathological microvascular growth under diabetic conditions [101]. MALAT1 knockdown ameliorates DR *in vivo* and regulates EC functions (cell proliferation, migration, and tube formation) *in vitro* via cross-talk between MALAT1 and the p38 MAPK signaling pathway [101]. Michalik et al. [111] found that MALAT1 levels are significantly increased by hypoxia and controlled by a phenotypic switch in ECs. Silencing of MALAT1 induces a promigratory response and increases basal sprouting and migration, whereas proliferation of ECs is inhibited. *In vivo* studies have confirmed that genetic ablation of MALAT1 inhibits the proliferation of ECs and reduces neonatal retina vascularization. Pharmacological inhibition of MALAT1 reduces blood flow recovery and capillary density after hindlimb ischemia. In conclusion, the knockdown of MALAT1 can tip the balance from a proliferative to a migratory EC phenotype *in vitro*; its genetic deletion, or pharmacological inhibition, reduces vascular growth *in vivo*. Puthanveetil et al. [112] investigated whether MALAT1 can regulate inflammatory pathways that involve inflammatory cytokines in diabetes. The increased levels of MALAT1, induced by HG, are associated with a parallel increase in serum amyloid antigen 3 (SAA3), an inflammatory ligand and target of MALAT1, and are further accompanied by an increase in the inflammatory mediators TNF-α and IL-6. Thus, MALAT1 participates in inflammatory activation by up-regulating TNF-α and IL-6 via SAA3 in ECs under hyperglycemic conditions.

#### MEG3

Maternally expressed gene 3 (*MEG3*) is a noncoding transcript belonging to the imprinted DLK1-MEG3 locus on chromosome 14q32.3 in humans. MEG3 is expressed in many normal tissues [113,114], but its expression is lost in several human tumors and tumor cell lines. The *MEG3* gene region, on chromosome 14q32.2, alters susceptibility to type 1 diabetes [102]. Epigenetic regulation of the DLK1-MEG3 miRNA cluster is altered in human type 2 diabetic islets [115]. This evidence implies a potential role of MEG3 in the pathological processes of DM. Qiu et al. [116] showed that the MEG3 expression level was significantly down-regulated in the retinas of STZ-induced diabetic mice and ECs under conditions of HG and oxidative stress. Silencing of MEG3 aggravated retinal vessel dysfunction *in vivo*, as shown by severe capillary degeneration, increased microvascular leakage, and inflammation. The knockdown of MEG3 also contributes to REC proliferation, migration, and tube formation *in vitro*. The roles of MEG3, in ECs, are mediated by the activation of the PI3K-Akt signaling pathway. Thus, up-regulation of MEG3 may serve as a therapeutic strategy for treating diabetes-related microvascular complications.

#### SOX2OT

SOX2 overlapping transcript (SOX2OT) is an lncRNA localized on human chromosome 3q26.33. Within one of its introns, SOX2OT holds the single-exon *SOX2* gene, overlapping in the same transcriptional orientation [117]. In humans, SOX2OT is highly enriched in the brain [103]. The retina and optic nerve are part of the brain; the eye and the brain share a number of common features [118]. Thus, SOX2OT may also be involved in the regulation of retinal neural function, thereby affecting retinal neurodegeneration. Li et al. [119] demonstrated that SOX2OT expression is significantly down-regulated in the retinas of STZ-induced diabetic mice, and in the RGCs, under HG or oxidative stress. SOX2OT knockdown protects RGCs against HG-induced injury *in vitro*, reversing the decreased cell viability and increased cell apoptosis induced by HG. Moreover, SOX2OT knockdown is neuroprotective in diabetes-related retinal neurodegeneration *in vivo*, playing an antioxidative role via regulation of nuclear factor erythroid 2 related factor 2/heme oxygenase-1 (NRF2/HO-1) signaling activity *in vitro* and *in vivo*. These results indicate that SOX2OT may be a promising therapeutic target for the prevention and treatment of diabetes-induced neural retinal neurodegeneration.

#### RNCR3

Retinal ncRNA3 (RNCR3), also known as LINC00599, was initially identified as an lncRNA expressed during mouse retinal development. RNCR3 was reported to be involved in neuronal and oligodendrocyte differentiation [104], and in atherosclerosis-related vascular dysfunction [105]. Emerging evidence indicates that retinal neurodegeneration, including neural apoptosis and reactive gliosis, is an important pathological feature of DR. Liu et al. [120] demonstrated that RNCR3 knockdown significantly inhibits retinal reactive gliosis. HG significantly up-regulates the expression of RNCR3 in Müller cells. Inhibition of RNCR3 leads to a marked reduction in the release of several cytokines, including IL-2, IL3, IL-4, IL-5, IL-9, IL-13, IL-17, MCP-1, VEGF, and TNF-α. RNCR3 knockdown alleviates DM-induced retinal neurodegeneration *in vivo*, as shown by the reduction in apoptotic retinal cells and improved visual function. RNCR3 knockdown also decreases the viability and proliferation of Müller glial cells and reduces the expression of glial reactivity related genes, including *GFAP* and vimentin, *in vitro*. These results indicate that RNCR3 knockdown inhibits retinal glial reactivity and prevents HG-induced retinal neurodegeneration.

Consistent with the previous study, Shan et al. [121] also confirmed that RNCR3 is significantly up-regulated, under Hg stress, in RF/6A cells, retinas of diabetic mice, and FVMs from PDR patients. RNCR3 knockdown significantly alleviates DM-induced retinal microvascular leakage *in vivo* and inhibits migration and tube formation by RF/6A cells under HG stress. According to these results, RNCR3 inhibition may be a treatment option for the prevention of DR-related retinal abnormalities.

#### ANRIL

Antisense RNA to INK4 locus (ANRIL) is important in cardiovascular diseases, type 2 diabetes, glaucoma, intracranial aneurysm, and cancers [122–125]. ANRIL consists of 19 exons, spanning 126.3 kb and producing a 3.8-bp RNA. It is situated in the *p15/CDKN2B-p16/CDKN2A-p14/ARF* gene cluster [126]. ANRIL may exert direct transcriptional effects, or play an indirect role, as a recruiter of chromatin remodeling complexes, such as polycomb repressor complex 2 (PRC2), to specific genomic loci [127,106]. Overexpression of ANRIL has also been shown to regulate the histone acetylase p300 [128]. Thomas et al. [129] demonstrated that HG and diabetes cause ANRIL up-regulation in HRECs and in the retina of diabetic animals, which is accompanied by an up-regulation of VEGF. The overexpression of VEGF is prevented by ANRIL siRNA transfection, showing a direct regulatory relationship. *In vivo* studies, using ANRIL knockout mice, have revealed that diabetes-induced up-regulation of retinal VEGF, and increased retinal microvascular permeability, are prevented by the down-regulation of ANRIL. Mechanistic studies found that ANRIL up-regulates VEGF via PRC2 components and p300 in glucose-exposed ECs and in the retinal tissue of diabetic animals. In summary, the regulatory effect of ANRIL on VEGF is mediated by interaction of ANRIL with components of the PRC2 complex, and histone acetylase p300, under diabetic conditions.

#### BDNF-AS

The lncRNA brain-derived neurotrophic factor antisense (BDNF-AS) is a natural antisense RNA of BDNF. BDNF-AS is naturally expressed in various human tissues, and may possibly have neural functions that are opposite to those of BDNF [130]. BDNF is expressed at low levels in animal models of, and patients with, DR [107,108]. The effects of BDNF-AS on apoptosis, in the progression of DR, were first investigated by Li et al. [131]. BDNF and BDNF-AS are inversely regulated by D-glucose-induced apoptosis, in which BDNF is significantly down-regulated, and BDNF-AS is up-regulated, by 50 mM D-glucose in ARPE-19 cells. Down-regulation of BDNF-AS ameliorates glucose-induced apoptosis in ARPE-19 and inversely up-regulates BDNF at both RNA and protein levels. BDNF-AS can directly target the binding sequence of BDNF, and regulate BDNF-associated cytokines, such as TNF-α and ILs [132]. Moreover, silencing of BDNF reverses the protective effect of BDNF-AS down-regulation on glucose-induced apoptosis; however, inhibition of BDNF does not completely reverse the protective effect of BDNF-AS down-regulation on apoptosis. Thus, BDNF-AS knockdown can protect retinal cells under diabetic conditions via BDNF-associated pathways, and may provide a new therapeutic target for the treatment of diabetes-associated retinal degeneration.

## Interactions between miRNAs and lncRNAs in DR

### MIAT–VEGF cross-talk occurs via competition for *miR-150-5p* binding in ECs

Recently, a hypothesis on ceRNA proposed that mRNAs and lncRNAs communicate with, and co-regulate each other. Thus, lncRNAs can act as miRNA sponges, regulating the miRNAs available for target mRNA binding [133]. The ceRNA may exhibit gene regulatory properties by competing with the mRNA for a shared set of miRNAs, thereby regulating the translation of their antagonist [134]. In their study, Yan et al. [98] put forward a model that includes lncRNA-MIAT, *miR-150-5p*, and *VEGF* mRNA. In diabetic rats, hyperglycemia significantly up-regulates MIAT levels; *miR-150-5p* knockdown can further increase MIAT levels. MIAT and *miR-150-5p* are coexpressed in the nuclei of RF/6A cells. Furthermore, *miR-150-5p* regulates MIAT in the nucleus in an Ago2-dependent manner. Additionally, MIAT overexpression significantly increases the level of VEGF, which is the target of *miR-150-5p* and is gradually reduced when *miR-150-5p* is up-regulated. In conclusion, during angiogenesis, MIAT can bind to the same site as *miR-150-5p*, thus alleviating the effect of *miR-150-5p* repression and up-regulating the level of VEGF. MIAT functions as a ceRNA and forms a feedback loop with VEGF, while *miR-150-5p* regulates EC function in response to HG.

### MIAT-*miR-29b*-Sp1 counteraction loop in HG-treated Müller cells

Sp1 expression is directly targetted by *miR-29b*, which is bound to the *miR-29b* promoter and represses the expression of *miR-29b* [135]. Concurrently, *miR-29b* inhibits the transcription of Sp1 [136]. In a new study, the expression of Sp1 was significantly increased in HG-induced rat rMC-1, which was accompanied by the up-regulation of MIAT and the down-regulation of *miR-29b* [99]. MIAT knockdown significantly reverses the low expression of *miR-29b*, and high expression of Sp1, induced by HG. These results indicate that MIAT can regulate the expression of *miR-29b* and Sp1 via harboring of *miR-29b*. The suppression of MIAT reverses the significant decrease in cell survival rate and increased cellular apoptosis induced by HG. Moreover, *miR-29b* knockdown significantly reverses the effects of cell survival and apoptosis mediated by MIAT suppression, which indicates that *miR-29b* knockdown interferes with the protective function of MIAT suppression. Thus, MIAT-controlled cellular apoptosis in DR may partly occur through absorption of *miR-29b* and inhibition of its function, as well as via regulation of expression of Sp1.

### The RNCR3/KLF2/*miR-185-5p* regulatory network modulates function of RECs

RNCR3 can act as a ceRNA, forming a feedback loop with Kruppel-like factor 2 (KLF2) and *miR-185-5p* to regulate HUVEC and vascular smooth muscle cells (VSMC) [105]. This study aimed to determine whether *miR-185-5p* regulates the level of RNCR3 in RF/6A cells. They found that injecting a *miR-185-5p* mimic significantly decreases the expression of RNCR3 and KLF2 in RF/6A cells. Transfection of a *miR-185-5p* mimic significantly decreases cell viability and inhibits proliferation of RF/6A cells. RNCR3 knockdown significantly decreases the viability and proliferation of RF/6A cells, whereas KLF2 overexpression reverses these effects. Thus, the RNCR3/KLF2/*miR-185-5p* regulatory network is involved in the regulation of RF/6A cellular function.

## Conclusion and perspectives

DR is a complication of DM, with multiple underlying pathogenic mechanisms, stemming from a variety of risk factors. A better understanding of the molecular mechanisms will help identify novel and effective targets for diagnosis and therapy. In recent years, miRNAs and lncRNAs have gained the attention of researchers in many fields including cancer, chronic diseases, and related complications. A number of miRNAs have been investigated and identified in the pathogenesis of DR, and a growing number of lncRNAs are being explored and studied with respect to their biological functions.

**Figure 1:**
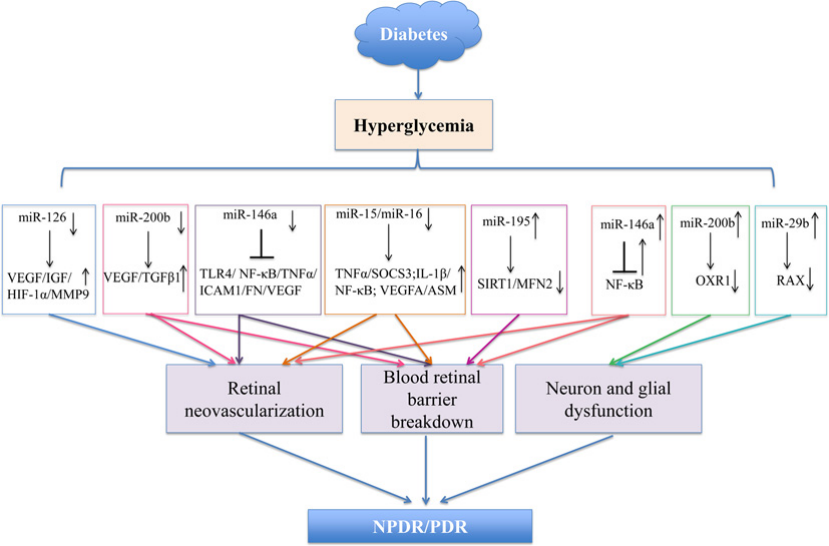
Major miRNAs associated with the pathogenesis of DR The main miRNAs involved in the progression, and contribution to DR, both *in vitro* and *in vivo* studies. The potential mechanisms of specific miRNAs (*miR-126, miR-200b, miR-146a, miR-195, miR-15/miR16*, and *miR-29b*) are demonstrated (see text for more details).

Presently, miRNAs represent a new powerful class of gene expression modulators in almost all human diseases. The involvement of miRNAs in the pathogenic mechanisms of DR was reported by microarray expression profiling studies, and specific roles of several aberrant miRNAs have been elucidated. The potential ability of a single miRNA to regulate several target genes, thus, influencing different molecular signaling pathways, has also been demonstrated. Amongst these, *miR-200b, miR-146a*, and *miR-126* have been most studied in the pathogenic processes of DR such as angiogenesis, inflammatory pathways, and oxidative stress (Figure 1). These miRNAs are considered potential biomarkers in the diagnosis and therapeutic targetting of DR. Although the number of studies is growing, the role of miRNAs, in a complex biological event such as DR, needs to be understood further; other miRNAs, and their target genes involved in this pathological event, are yet to be discovered.

In addition to miRNAs, lncRNAs have also been implicated in DR. However, the roles of lncRNAs in the pathogenesis of DR are far from being understood. Similarly, microarray analysis and RNA sequencing provide convenient and comprehensive approaches for identifying lncRNAs aberrantly expressed in DR. Moreover, the roles of specific lncRNAs, in the development of DR, are explored by investigating their involvement in targetting of different mRNAs and cross-talking with miRNAs. Most lncRNAs, mentioned in this review, were identified by consulting relevant studies about diseases that share the same etiology or pathogenesis. For example, ANRIL is significantly associated with increased susceptibility to type 2 DM [125]; thus, it is of no surprise that this aberrantly expressed lncRNA may be relevant in the molecular mechanisms underlying DR [129]. Systematic identification of lncRNAs, and a better understanding of their mechanisms of action, can pave the way for early diagnosis and improved therapeutics for DR. New candidate lncRNA genes, and their molecular mechanisms, remain to be explored.

As discussed in this review, miRNAs and most lncRNAs regulate specific protein activity, and may, thus, represent potential targets for drugs that are more refined and less toxic than conventional protein-targetting drugs. Intensive research will inspire new hypotheses about pathogenesis and will lead to novel clinical applications. Understanding the roles of these molecules in DR will help develop novel strategies to effectively treat this disease and decrease the rate of patients developing blindness due to progression of retinopathy.

## Footnotes

* miR-29c* was produced from the opposite arm of the precursor, while miR-29c was the predominant product.

## Abbreviations

ADA: adenosine deaminase
AGA: amadori-glycated albumin
Ago: argonaute
AGT: angiotensinogen
AKT3: serine/threonine-protein kinase 3
Ang-2: angiopoietin-2
ANRIL: antisense RNA to INK4 locus
ASM: acid sphingomyelinase
BDNF-AS: brain-derived neurotrophic factor antisense
BRB: blood–retinal barrier
CARD10: caspase-recruitment domain containing protein 10
ceRNA: competing endogenous RNA
CNV: choroidal neovascularization
DAXX: death domain associated protein
DM: diabetes mellitus
DR: diabetic retinopathy
EC: endothelial cell
ECM: extracellular matrix
ERK: extracellular signal regulated kinase
EV: extracellular vesicle
FN: fibronectin
FVM: fibrovascular membranes
GPCR: G-protein-coupled receptor
GWAS: genome-wide association study
HG: high glucose
HIF-1α: hypoxia-inducible factor-1 α
HREC: human retinal EC
ICAM-1: intercellular adhesion molecule-1
IGF: insulin-like growth factor
IGF1R: IGF-1 receptor
INL: inner nuclear layer
IRAK1: IL-1 receptor associated kinase 1
KLF2: Kruppel-like factor 2
lncRNA: long noncoding RNA
MALAT1: metastasis-associated lung adenocarcinoma transcript 1
MAPK: mitogen-activated protein kinase
MEG3: maternally expressed gene 3
MFN2: mitofusin 2
MIAT: myocardial infarction associated transcript
MMP-9: matrix metalloproteinase 9
MSC: mesenchymal stem cell
MyD88: myeloid differentiation primary-response protein-88
ncRNA: noncoding RNA
NPDR: non-proliferative DR
NV: neovascularization
OIR: oxygen-induced retinopathy
PDR: proliferative DR
PKR: RNA-dependent protein kinase
PRC2: polycomb repressor complex 2
pri-miRNA: primary miRNA
PVD: proliferative vitreoretinal disease
RAX: PKR-associated protein X
REC: retinal EC
RGC: retinal ganglion cell
RISC: RNA-induced silencing complex
rMC-1: retinal Müller cell
ROS: reactive oxygen species
RPE: retinal pigment epithelial
SAA3: serum amyloid antigen 3
SIRT1: silent information regulator protein 1
SOX2OT: SOX2 overlapping transcript
STAT3: signal transducer and activator of transcription 3
STZ: streptozotocin
TGF-β: transforming growth factor β
TLR: toll-like receptor
TNF: tumor necrosis factor
TRAF6: TNF receptor associated factor 6
VEGF: vascular endothelial growth factor
VSMC: vascular smooth muscle cell
